# Signal transduction inhibitors for the treatment of rheumatoid arthritis

**DOI:** 10.1186/ar3593

**Published:** 2012-02-09

**Authors:** Yoshiya Tanaka, Kunihiro Yamaoka

**Affiliations:** 1 The First Department of Internal Medicine, University of Occupational and Environmental Health, Japan, Kitakyushu, Japan

## 

Rheumatoid arthritis (RA) is a representative autoimmune disease characterized by chronic and destructive inflammatory synovitis. The multiple cytokinesand cell surface molecules play a pivotal role in the pathogenesis of RA and binding of these molecules to their ligands on the cell surfaceinduce various signal intracellular transduction including phosphorylation of kinase proteins. The tyrosine kinase is the first intracellular signals to be phosphorylated and 14 tyrosine kinases are known to be involved in RA. Among them, members of Janus kinase (Jak) familyare essential for the signaling pathways of various cytokines and are implicated in the pathogenesis of RA. An orally available Jak3 inhibitor tofacitinib is currently in clinical trials for RA with satisfactory effects and acceptable safety [1,2]. A phase 2 double-blinded study wascarried out to investigate the efficacy and safety of tofacitinib in Japanese patients with active RA andinadequate responseto methotrexate (MTX). A total of 140 patients were randomized to tofacitinib 1, 3, 5, 10 mg, or placebotwice daily and ACR20 response rates at week 12, a primary endpoint, was significant for all tofacitinib treatment groups [3]. Thus, tofacitinib in combination with MTX was efficacious and had a manageable safety profile and tofacitinib 5 and 10 mg twice a day appear suitable for further evaluation to optimize their potential for the treatment of RA. Although the mode of action of tofacitinib has remain unclear, we clarified thatthe inhibitory effects of tofacitinib could be mediated through the suppression of IL-17 and IFN-γ production and proliferation of CD4^+ ^T cells, presumably Th1 and Th17 cells by in vitro experiments. We next conducted a treatment study in the SCID-HuRAg mice, an RA animal model utilizing SCID mice implanted with synovium and cartilage from patients with RA and tofacitinib was administered via an osmotic mini-pump. Tofacitinib decreased serum levels of human IL-6 and IL-8 in the mice and reduced invasion of the synovial tissue into the implanted cartilage as well as accumulation of immune cells in the synovium.Taken together, orally available low molecular weight products such as tofacitinib targeting intracellular signaling molecules, would provide enormous power and flexibility in the treatment of RA.
